# Séroprévalence de l’AgHBs chez la femme enceinte dans le centre du Maroc

**DOI:** 10.11604/pamj.2016.24.187.9849

**Published:** 2016-07-01

**Authors:** Mohammed Sbiti, Hanane Khalki, Imane Benbella, Lhoussaine louzi

**Affiliations:** 1 Service de Microbiologie, Hôpital Militaire Moulay Ismail, Faculté de Médecine et de Pharmacie de Fès, Maroc; 2 Pôle de Biologie et Pharmacie, Hôpital Militaire Moulay Ismail, Meknès, Maroc

**Keywords:** HBsAg, seroprevalence, viral load, prevention of HBV vertical transmission, HBsAg, seroprevalence, viral load, prevention of HBV vertical transmission

## Abstract

La transmission materno-fœtale du virus de l'hépatite B (VHB) est un problème qui préoccupe toujours les autorités sanitaires à travers le monde et suscite la mise en place de mesures préventives renforcées. Le statut réplicatif du virus chez la mère séropositive, évalué par la positivité de l'AgHbe et le taux de la charge virale, conditionne le risque de contamination qui est plus important en période péri-natale. Les mères porteuses chroniques du VHB constituent un véritable réservoir de la transmission verticale de cette infection. Nous avons étudié, à travers cette enquête sur 1120 femmes enceintes Marocaines, la séroprévalence de l'AgHBs qui était de 2,35%, dans le but d'alimenter les données nationales sur ce sujet. Parmi ces femmes séropositives pour l'AgHBs, 79,1% avaient un AgHBe négatif et ont bénéficié d'une recherche moléculaire qui s'est révélée positive dans 89,4% des cas. La vaccination de la femme en âge de procréer est l'un des piliers majeurs de la prévention de la transmission verticale du VHB, seulement 2,4% de nos patientes étaient vaccinées. Ceci relève l'intérêt du dépistage au cours de la grossesse, qui doit se focaliser sur la sensibilisation à la vaccination de femmes séronégatives, et le suivi par biologie moléculaire des mères séropositives dans le but d'instaurer un traitement prophylactique adéquat.

## Abstract (en)

Maternal-fetal transmission of hepatitis B virus (HBV) is a global public health problem leading to the implementation of strengthened prevention measures. Viral replication in HIV-positive mother, assessed based on HBeAg positivity and the rate of viral load, determines the contamination risk which is very important during perinatal period. Mothers with chronic HBV are a real reservoir of vertical transmission of this infection. This is a prospective investigation of 1120 pregnant Moroccan women aiming to study the seroprevalence of HBsAg which was 2.35%, in order to supply the national evidence on this topic. Among these HIV-positive women who were HBsAg positive, 79.1% were HBeAg negative and underwent molecular research that was positive in 89.4% of cases. Immunization of women of childbearing age is one of the major pillars of prevention of HBV vertical transmission. Only 2.4% of our patients were vaccinated. This highlights the role of screening during pregnancy, which should focus on raising awareness about the importance of immunization in HIV-negative women and of monitoring using molecular biology tools in HIV-positive mothers in order to establish an appropriate prophylactic treatment.

## Introduction

L'infection par le virus de l'hépatite B (VHB) est un des principaux problèmes de santé publique à l'échèle nationale et mondiale. La gravité de l'hépatite B est liée au risque de passage à la chronicité et d'exposer les malades à la cirrhose et au cancer hépatique, ce risque est d'autant plus important que l'infection survient à un âge précoce, notamment en cas de contamination néonatale [1]. L'organisation mondiale de la santé (OMS) estime à deux milliards le nombre de personnes ayant été exposées à ce virus et près de 10 à 30 millions de nouvelles contaminations par an, avec près d'un million de décès chaque année. Le réservoir du VHB est représenté par le nombre important de porteurs chronique à travers le monde et qui est estimé à plus de 350 millions [2]. Le Maroc fait partie des zones d'endémicitémoyenne inférieure pour le VHB avec un taux de portage chronique de l'AgHBs variant de 2 à 4 % dans la population générale [3]. A notre connaissance, il y a très peu de données nationales qui sont disponibles quant à la séroprévalence de l'infection par le VHB chez la femme enceinte. La transmission matérno-fœtale du VHB représente un maillon essentiel d'entretien de l'infection, surtout dans les pays de forte endémicité. Ce risque est estimé à 90-100% si l'antigène HBe (AgHBe) est détecté dans le sérum maternel. La présence d'AgHBe a également été associée à un risque élevé d'échec de la prévention néonatale. Toutefois, même en l'absence d'AgHBe (mutants pré-core) le risque de transmission du VHB existe et l'interprétation doit tenir compte de la virémie [4]. L'objectif de ce travail est d'étudier la séroprévalence de l'infection par le VHB chez une population de femmes enceintes Marocaines, et de mettre le point sur le risque de transmission verticale.

## Méthodes

Il s'agit d'une étude transversale, observationnelle sur la séroprévalence de l'Ag HBs chez les femmes enceintes au niveau l'Hôpital Militaire Moulay Ismail de Meknès du Centre Hospitalier Universitaire Hassan II de Fès. Depuis janvier 2014 à décembre 2015, 1120 sérums ont été recueillis par le laboratoire d'immuno-sérologie provenant des femmes enceintes qui ont consulté au service de gynécologie de l'hôpital, au niveau de l'infirmerie de garnison et chez différents médecins de la ville à titre externe. Des prélèvements sanguins ont été effectués chez 1120 femmes enceintes dans le cadre de bilan de grossesse pour dépister l'AgHBs. Les prélèvements de sang ont été recueillis stérilement dans des tubes secs. Les sérums obtenus après centrifugation ont été aliquotés en deux parties, l'une pour la sérologie et l'autre pour un éventuel contrôle ayant été conservé à -20^°^ C jusqu'à utilisation. En plus du prélèvement sanguin, des données épidémiologiques concernant l'âge, le terme de la grossesse, la notion de vaccination antérieure contre le VHB, les antécédents médicaux, chirurgicaux et obstétricaux ont été collectées pour 740 femmes sur les 1120 étudiées. La participation à cette étude était donc entièrement volontaire et à l'aide d'une fiche d'exploitation préétablie. Le dépistage de l'AgHBs a été réalisé par technique immuno-enzymatique de type Elisa (Enzyme LinkedImmunoSorbentAssay) de troisième génération par le Kit « Monolisa^™^ HBs Ag ULTRA» (Bio-Rad). Tous les sérums positifs au premier test ont subis un deuxième test de confirmationde la positivité de l'AgHBs. Une recherche de l'AgHBe, des anticorps anti-HBe et des anticorps IgM anti-HBc a été faite par technique chimiluminescence automatisée (ARCHITECT /Abbott). L'analyse moléculaire pour les différentes patientes séropositives a été réalisée à titre externe dans différents laboratoires vue la non disponibilité d'automate nécessaire pour la technique de PCR dans notre laboratoire durant cette période.

## Résultats

L'âge moyen des femmes enceintes concernées par l'étude était de 28 ans +/- 6 ans avec des extrêmes allant de 17 à 43 ans. L'âge moyen de la grossesse en cours était de 17.1 +/- 4.7 semaines d'aménorrhées (SA). Toutes ces femmes avaient déjà eu au moins une grossesse dans 66,2% des cas (490/740) avec une moyenne de 2,3 [0-7]. Le taux de scolarisation était de 26,1%. Presque toutes les gestantes (99,6 %) ignoraient antérieurement leur statut sérologique vis-à-vis du VHB. La notion d'une vaccination antérieure contre le VHB a été rapportée dans uniquement 2,4% des cas (18/740) de nos patientes. Parmi les femmes enceintes dépistées, 2,35% (24 /1021) se sont révélées positives pour l'AgHBs; elles avaient toutes déjà eu au moins une grossesse dans 75% des cas (18/24). Parmi celles avec AgHBs (+), deux (8,3%) connaissaient antérieurement leur statut sérologique vis-à-vis du VHB. Sur ces 24 femmes positives pour l'AgHBs, 79,1% (19/24) avaient un profil AgHBe (-), AcAntiHBe (+); et cinq femmes étaient positives pour l'AgHBe. L'analyse moléculaire a pu être réalisée pour toutes les femmes AgHBe (-). L'ADN viral était détectable dans 89,4% des cas testés (17/19); seul deux sérums (10,5%) se sont révélés négatifs. Pour les sérums positifs par PCR, la charge virale était variable avec des titres allant de 34 à 10^8^copies/ml. Sur les 17 sérums où l'ADN était détectable, une charge virale modérée (< 10^4^ copies/ml) a été détectée dans 76, 4% des cas (13/17); parmi ces derniers, 11,7% (2/17) avaient un titre variant entre 34 et 200 copies/ml. Le titre d'ADN viral était compris entre 10^4^ et 10^8^ copies/ml dans 17,6% (n = 3 /17) (**Tableau 1**).

**Tableau 1:** Résultats de la quantification de l’ADN viral pour les sérums AgHBe(–) et positifs par PCR

Charge virale copies/ml	34 à 10^4^		10^4^ à 10^8^		> 10^8^		Total	
	N	%	N	%	N	%	N	%
Sérums AgHBe (–) et positifs par PCR	13	76,6	3	17,6	1	5,8	17	100

## Discussion

Le virus de l'hépatite B est le virus le plus fréquent à l'échelon mondial, et le plus contagieux, posant un problème extrêmement préoccupant de prévention de la transmission materno-foetale. Si dans les pays industrialisés, c'est une infection surtout liée au sexe ou au sang, en Afrique, la contamination mère-enfant en période péri- et néo-natale du virus est le mode majeur de transmission horizontale par contact très étroit dès l'enfance [5]. Le VHB sévit à l'état endémique avec une prévalence différente selon les régions du globe. L'AgHBs est un bon marqueur de dépistage du VHB dans une population, car il révèle soit un statut de VHB aiguë ou un statut de porteur chronique du VHB. Au Maroc, peu d'études épidémiologiques ont été faites pour étudier la séroprévalence de l'AgHBs chez la femme enceinte, mais on estime que la prévalence de l'AgHbs dans la population générale est de 1,5% [6]. C'est pour cette raison que l'objectif principal de cette étude était d'estimer le niveau de l'infection par le VHB chez des femmes enceintes Marocaines par le dépistage d'un nombre élevé de femmes pour l'AgHBs. Notre étude trouve une séroprévalence de l'AgHBs de 2,35% chez la femme enceinte dans la région du centre du Maroc, parmi elles 20,9% avaient un profil AgHBe+. Une étude prospective sur 156 femmes enceintes s'étalant sur trois mois dans la région de Fès a trouvé un taux moins important de 1,3% [7]. Nos taux sont plus bas par rapport à nos voisins méditerranéens, telles que la Tunisie, la Grèce et la Turquie avec 3 à 4% [8], 3,8 % [9] et 3,8% [10] respectivement. Ils sont cependant, plus élevés par rapport aux pays de la rive Nord, tels que la France 0.65% [11] ou l'Espagne 0.4% [12]. Des prévalences beaucoup plus élevées ont été notées en Égypte 8% [13], en Libye 11.1% [14], en Mali 15,5% [5] et en Côte d'ivoire 18% [15] (Tableau 2).

**Tableau 2:** Revue de la littérature sur la séroprévalence de l’AgHBs chez la femme enceinte dans différentes séries

Série (Auteur-année Pays)	Total	Pourcentage AgHBs+
Hannachi -2008 [8] Tunisie	2303	3-4
Panagopoulos -2004 [9] Grèce	5497	3,8
Kuri-1996 [10] Turquie	5366	3,8
Denis-2004 [11] France	22859	0,65
Gutierrez-2004 [12] Espagne	2929	0,4
Harder-2011 [16] Danemark	140376	0,26
El Nawawy-1996 [13] Egypte	150	8
Christie-1976 [14] Libye	293	11,1
Sidibe-2001 [5] Mali	829	15,5
Lahoues-1998 [15] Côte d’ivoire	-	18
Sekkat- 2010[7] Maroc	156	1, 3
**Notre série**	**1120**	**2,35**

L'immigration joue un rôle important dans les variations des taux de prévalence de l'infection par VHB dans les pays d'accueil, du fait que les femmes enceintes originaires de régions à forte endémicité telle que l'Asie ou l'Afrique expriment des taux de prévalence beaucoup plus élevés. Ceci étant bien illustré dans une étude danoise [16] qui rapporte que la prévalence de l'AgHBs chez les femmes enceintes quelques soient leurs origines ethniques est de 0,26% (AgHBe 17,5%) alors qu'elle est de 14% pour les femmes originaires de l'Asie de Sud-Est. La faible prévalence de l'AgHBe dans notre étude ne l´empêche pas d´identifier un profil sérologique de l´infection par un mutant virus pré C ou prénucléocapside, identifié par un portage de l´AgHBs et une charge virale importante de l´ADN chez ces femmes avec un AgHBe négatif. Le risque de transmission materno-fœtale est variable selon le statut de la réplication du VHB chez la mère, qui est lui aussi corrélé à la positivité de l'AgHBe. Ce risque est de 90% quand l'AgHBe est positif, alors qu'il n'est que de 10 à 20% quand l'AgHBe est négatif [17]. La grossesse n´augmente pas le risque d´évolution d'une hépatite aigue vers l'hépatite fulminante, et ne semble pas, non plus, augmenter le risque de développer une infection chronique après primo-infection [8]. Chez un sujet dont le statut sérologique antérieur n´était pas connu, la recherche de l'anti-HBc de type IgM trouve son intérêt pour la distinction entre une primo infection et une hépatite aiguë par réactivation du VHB qui peut être observée pendant la grossesse. Quant à l l´infection par le VHB, elle augmenterait la fréquence du diabète gestationnel, la menace d´accouchement prématuré, le petit poids à la naissance et l'avortement spontané sans avoir par ailleurs d'effet tératogène [18].

Chez le nouveau né, n'ayant pas bénéficié d'une sérovaccination prophylactique, une hépatite fulminante peut être observée [19]. Le passage à la chronicité d'une hépatite virale B (persistance de l'AgHBs pendant plus de six mois sans IgM anti-HBc), survient après une infection aigue dans 90 à 95% des cas d'infection péri-natales, dans 25 à 50% des cas chez les enfants entre un et cinq ans et dans 5 à 10% des cas chez l'adulte ou chez l'enfant de plus de cinq ans [18]. Avec un taux non négligeable supérieur ou égal à vingt pour cent des infections chroniques qui évolueront vers une cirrhose. La quantification de la charge virale durant la grossesse permet non seulement d'évaluer le risque de transmission du VHB afin de mettre en œuvre une sérovaccination rapide et efficace dès la naissance, mais également de prévoir un traitement antiviral maternel au cours du troisième trimestre de grossesse lorsque la virémie est élevée. Dans notre série 89,4% des femmes positives pour l'AgHBs, mais négatives pour l'AgHBe avaient un ADN détectable par PCR avec des charges virales supérieures à 10^4^ copies par millilitre pour près de 23,4% d'entre elles. Ce statut AgHBe négatif avec virémie élevée est associé à un risque de transmission verticale du virus non négligeable, et à un risque accru d'hépatites fulminantes chez le nouveau-né. Ce résultat démontre l'importance des techniques moléculaires pour évaluer le risque de transmission du VHB de la mère à l'enfant quel que soit le profil sérologique vis-à-vis de l'AgHBe, surtout dans notre pays où plus de 90% des porteurs chroniques sont infectés par un virus mutant au niveau du gène C [19].

Sur le plan prévention, une vaccination systématique des nourrissons contre le VHB a été instaurée à la naissance depuis 1999 dans le Programme National d'Immunisation (PNI) Marocain, le dépistage de l'antigène HBs (AgHBs) est devenu obligatoire au quatrième examen prénatal (6ième mois de grossesse).

Si la contamination anténatale ne peut être prévenue par une sérovaccination à la naissance, la contamination périnatale elle, bénéficie d'une sérovaccination efficace dès lors qu'elle est initiée le plus précocement possible. Chez les enfants nés de mères porteuses chroniques des AgHBs et AgHBe, il semble préférable d'utiliser dès la naissance une dose importante d'IgHBs (0,6 ml/kg) à renouveler au bout de trois semaines pour tenir compte de la cinétique des immunoglobulines et d'utiliser comme chez les prématurés, un schéma vaccinal de quatre doses (naissance, j21, j60, 12 mois) de vaccin anti-VHB titrant à 10 µg. Une surveillance sérologique attentive dans les premiers mois de vie (naissance, trois semaines de vie et un mois après la sérovaccination initiale) paraît souhaitable en raison du pourcentage important de mauvais répondeurs possibles [20]. Toutefois, dans notre contexte en raison de la non disponibilité des services d'obstétrique et de pédiatrie au sein de notre formation hospitalière, le suivi des nouveaux nés de mères séropositives n'a pas pu être réalisé.

## Conclusion

Actuellement, les tests de dépistage et les techniques de biologie moléculaire sont disponibles dans la majorité du territoire marocain, ce qui permet de réaliser le diagnostic et le suivi de l´infection par VHB. Une étude plus large devrait être entreprise pour ressortir la séroprévalence nationale de l´infection chez les femmes enceintes Marocaines. Devant l'ampleur de ce fléau mondial il est primordial insister sur les mesures préventives de la transmission périnatale par une sérothérapie dès la naissance voir même un traitement antiviral en fin de grossesse. Il est recommandé aussi de promouvoir la vaccination contre VHB et de contrôler le statut sérologique avant la grossesse. L'efficacité de la vaccination anti VHB induit l'apparition d'une concentration d'anticorps protectrice chez plus de 95% des nourrissons, des enfants et des jeunes adultes.

### Etat des connaissances actuelle sur le sujet

- Le Maroc fait partie des zones d'endémicité moyenne inférieure pour le VHB;
- Les mères porteuses du VHB constituent un véritable réservoir cette infection;
- le risque de contamination est conditionne parle taux de la charge virale.

### Contribution de notre étude à la connaissance

- La première séroprévalence de l'AgHBs dans la région du centre du Maroc chez la femme enceinte de 2,35%;
- Seulement 2,4% de nos patientes étaient vaccinées;
- 23,4% avaient un risque de transmission verticale du virus non négligeable.
